# Vitamin D and adipose tissue—more than storage

**DOI:** 10.3389/fphys.2014.00228

**Published:** 2014-06-24

**Authors:** Shivaprakash J. Mutt, Elina Hyppönen, Juha Saarnio, Marjo-Riitta Järvelin, Karl-Heinz Herzig

**Affiliations:** ^1^Department of Physiology, Institute of Biomedicine, University of OuluOulu, Finland; ^2^Biocenter of Oulu, University of OuluOulu, Finland; ^3^School of Population Health and Sansom Institute, University of South AustraliaAdelaide, SA, Australia; ^4^South Australian Health and Medical Research InstituteAdelaide, SA, Australia; ^5^Population, Policy and Practice, Institute of Child Health, University College LondonLondon, UK; ^6^Department of Surgery, Oulu University Hospital, University of OuluOulu, Finland; ^7^Unit of Primary Care, Institute of Health Sciences, University of Oulu, Oulu University HospitalOulu, Finland; ^8^Department of Children, Young People and Families, National Institute for Health and WelfareOulu, Finland; ^9^Department of Epidemiology and Biostatistics, and MRC-PHE Center for Environment and Health, School of Public Health, Imperial College LondonLondon, UK; ^10^Medical Research Center Oulu and Oulu University HospitalOulu, Finland

**Keywords:** 1,25-dihydroxycholecalciferol or calcitriol, vitamin D binding protein, gene regulation, adipose tissue, adipogenesis, secretion, adipokines

## Abstract

The pandemic increase in obesity is inversely associated with vitamin D levels. While a higher BMI was causally related to lower 25-hydroxyvitamin D (25(OH)D), no evidence was obtained for a BMI lowering effect by higher 25(OH)D. Some of the physiological functions of 1,25(OH)_2_D_3_ (1,25-dihydroxycholecalciferol or calcitriol) via its receptor within the adipose tissue have been investigated such as its effect on energy balance, adipogenesis, adipokine, and cytokine secretion. Adipose tissue inflammation has been recognized as the key component of metabolic disorders, e.g., in the metabolic syndrome. The adipose organ secretes more than 260 different proteins/peptides. However, the molecular basis of the interactions of 1,25(OH)_2_D_3_, vitamin D binding proteins (VDBPs) and nuclear vitamin D receptor (VDR) after sequestration in adipose tissue and their regulations are still unclear. 1,25(OH)_2_D_3_ and its inactive metabolites are known to inhibit the formation of adipocytes in mouse 3T3-L1 cell line. In humans, 1,25(OH)_2_D_3_ promotes preadipocyte differentiation under cell culture conditions. Further evidence of its important functions is given by VDR knock out (VDR^−/−^) and CYP27B1 knock out (CYP27B1 ^−/−^) mouse models: Both VDR^−/−^ and CYP27B1^−/−^ models are highly resistant to the diet induced weight gain, while the specific overexpression of human VDR in adipose tissue leads to increased adipose tissue mass. The analysis of microarray datasets from human adipocytes treated with macrophage-secreted products up-regulated VDR and CYP27B1 genes indicating the capacity of adipocytes to even produce active 1,25(OH)_2_D_3_. Experimental studies demonstrate that 1,25(OH)_2_D_3_ has an active role in adipose tissue by modulating inflammation, adipogenesis and adipocyte secretion. Yet, further *in vivo* studies are needed to address the effects and the effective dosages of vitamin D in human adipose tissue and its relevance in the associated diseases.

## Introduction

**Figure 1:**
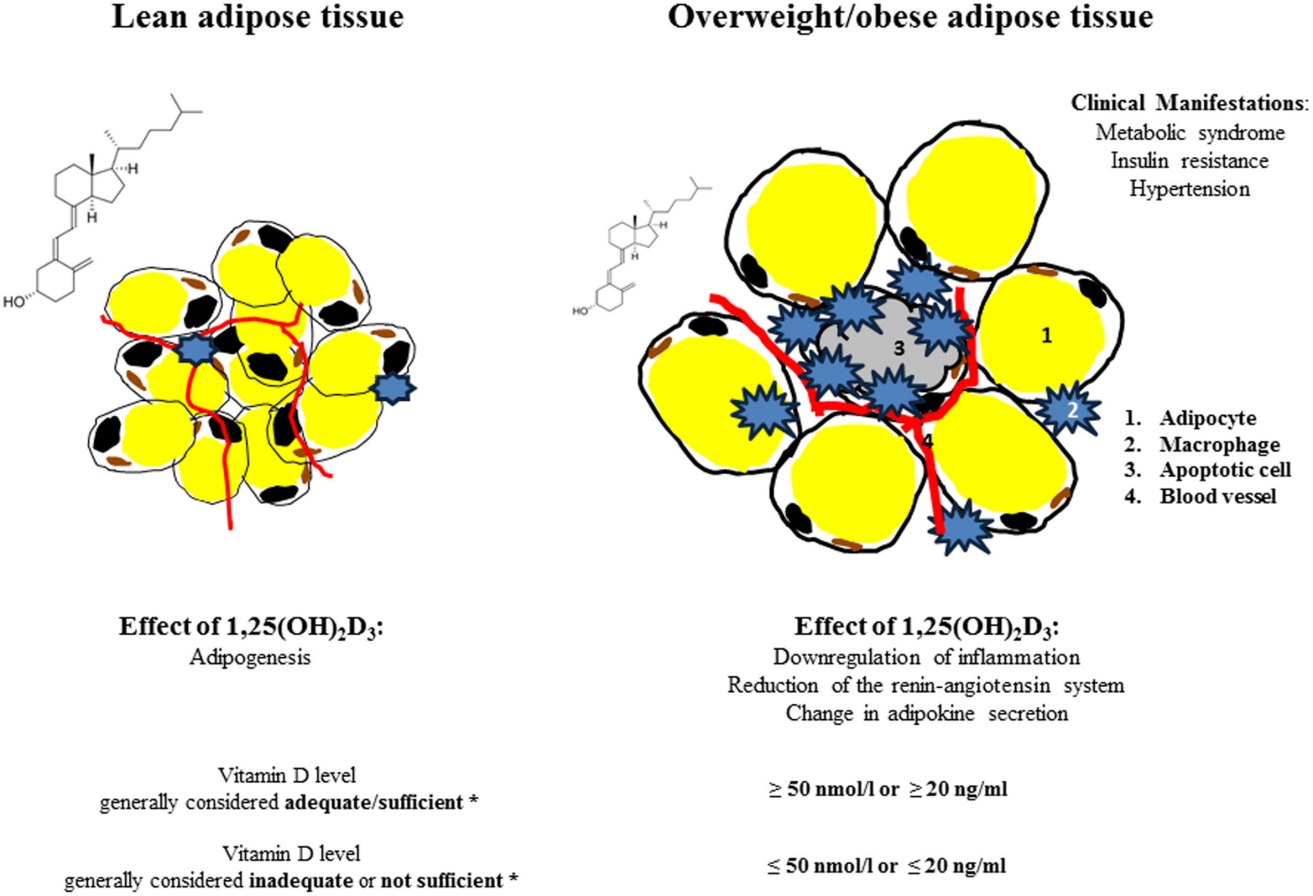
**Effect of 1,25(OH)_2_D_3_ on adipose tissue/lean and obese state**. ^*^Vitamin D levels (25(OH)D) according to the Institute of Medicine, Food and Nutrition Board. Dietary Reference Intakes for Calcium and Vitamin D. Washington, DC: National Academy Press, 2011.

Adipose tissue is no longer regarded as a simple storage organ since it has been convincingly shown that it secretes more than 260 different proteins/peptides (Lehr et al., 2012). Lean people have about 5 kg of adipose tissue, while in obese and severely obese individuals the adipose tissue/organ could amount to 50 kg or more (Frankenfield et al., 2001). Excess in adipose tissue has been attributed to a variety of diseases including cancer, diabetes, cardiovascular and neurodegenerative diseases and decrease in life expectancy (Adams et al., 2006; Despres and Lemieux, 2006; Kahn et al., 2006; Van Gaal et al., 2006). Adiposity is one of the most serious public health problems, associated with vitamin D insufficiency due to the decreased bioavailability of vitamin D_3_ (Wortsman et al., 2000). The Institute of Medicine (IOM) recommended 25-hydroxyvitamin D (25(OH)D) levels as reliable biomarker for assessment of Vitamin D status; currently values ≤50 nmol/l or ≤20 ng/ml are considered inadequate or not sufficient and values ≥50 nmol/l or ≥20 ng/ml as adequate or sufficient (Ross et al., 2011) (Figure 1). 25(OH)D levels have been determined by a variety of methods yielding different results. The National Institutes of Health’s Office of Dietary Supplements together with National Institute of Standards and Technology (NIST) therefore developed a standard reference material-972 (SRM-972) for accuracy of laboratory vitamin D measurements (Phinney et al., 2012). A recent study by the D-CarDia consortium employed a Mendelian randomization (MR) approach to establish causality and direction of the association between vitamin D status and obesity measured by body mass index (BMI) using information from 21 adult cohorts (up to 42,024 participants) (Vimaleswaran et al., 2013). The consortium found that a higher BMI was causally related to lower 25(OH)D; no evidence was obtained for a BMI lowering effect of higher 25(OH)D. However, the study did not provide insights into the cellular action of 1,25(OH)_2_D_3_ (1,25-dihydroxycholecalciferol or calcitriol). While the knowledge of the effects of 1,25(OH)_2_D_3_ as an essential hormone and transcription factor is further emerging, it is increasingly acknowledged that 1,25(OH)_2_D_3_ down regulates inflammatory responses in the adipose tissue. The anti-inflammatory effects of 1,25(OH)_2_D_3_ might have notable influences on population health and disease prevention, since inflammation is thought to be the underlying cause of a range of metabolic disorders (Hotamisligil, 2006; Huotari and Herzig, 2008; Vlasova et al., 2010).

## Vitamin D and adipogenesis

**Figure 2:**
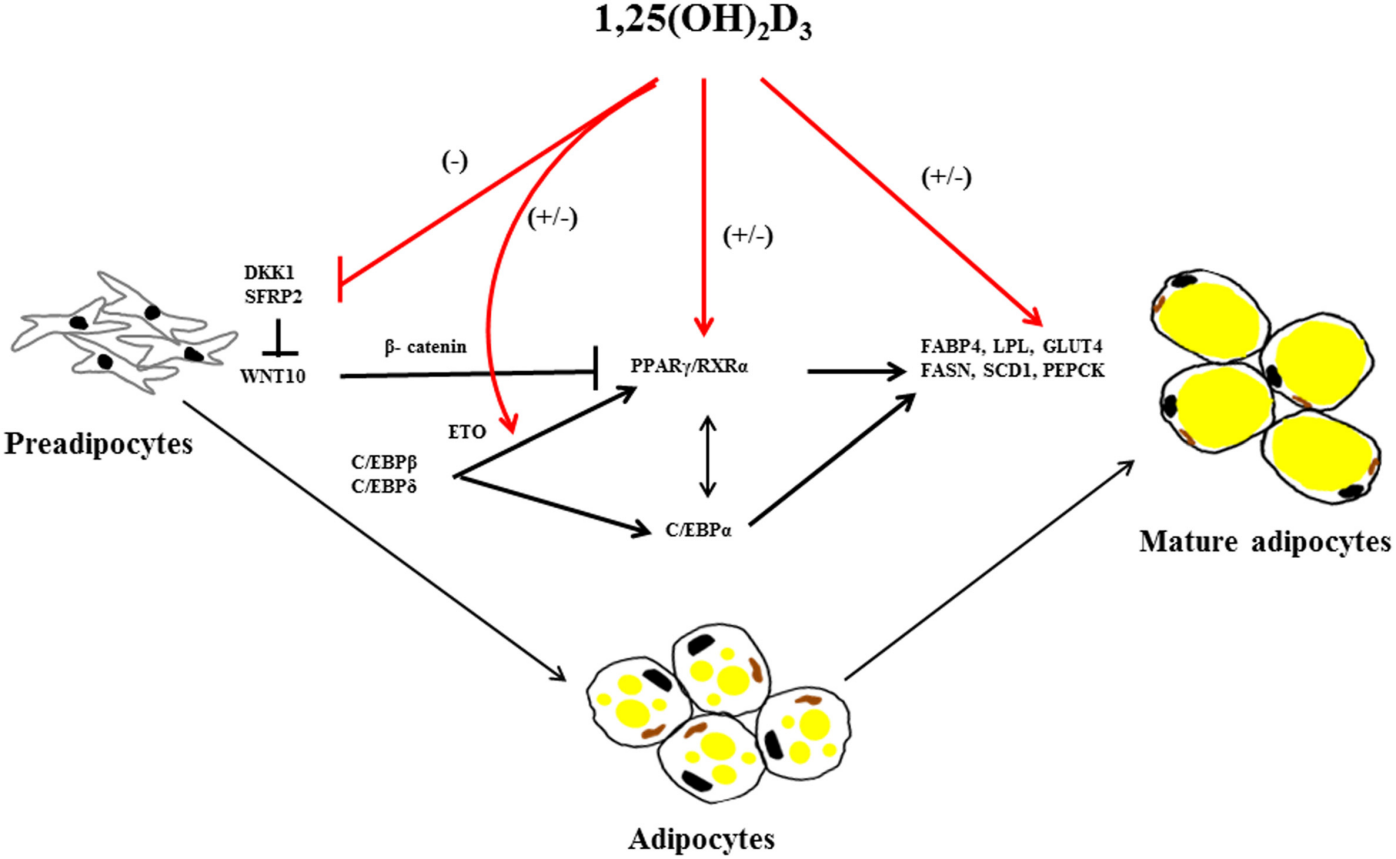
**Overview of 1,25(OH)_2_D_3_ on regulating factors in adipogenesis**. 1,25(OH)_2_D_3_ has both stimulating and inhibition effect on adipogenesis. 1,25(OH)_2_D_3_ suppress dickkopf1(DKK1) and secreted frizzled-related protein 2 (SFRP2) expression in mouse bone marrow stromal cells (BMSCs) there by suppressing adipogenic peroxisome proliferator-activated receptor γ/retinoid X receptor complex (PPARγ/RXRα) mediated by stabilization of β-catenin through wingless-type MMTV integration site family, member 10 (WNT10) inhibition. Furthermore, 1,25(OH)_2_D_3_ regulates several adipogenic mediators during differentiation, proliferation and maturation of mesodermal cells into adipocytes including *CCAAT/enhancer-binding proteins α, β, and γ (C/EBPα, β, and γ), C/EBPβ corepressor eight twenty-one (ETO), PPARγ/RXRα, fatty acid binding protein 4 (FABP4), lipoprotein lipase (LPL), fatty acid synthase (FASN), stearoyl-coA desaturase-1 (SCD1), glucose transporter type 4 (GLUT4) and phosphoenolpyruvate carboxykinase (PEPCK)*. The arrows in the figure indicate activation and blunted line indicate inhibition. The red lines and (+/−) indicate stimulatory or inhibitory effect of 1,25(OH)_2_D_3_ on adipogenesis dependent on the species or cellular systems studied.

Adipose tissue expansion is a remarkable process characterized by the enlargement of adipocyte size known as hypertrophy and by the increase in the number of adipocytes known as hyperplasia, which is more strongly associated with severity of obesity (Arner and Spalding, 2010). Both processes emerge through sequential stages of differentiation to form mature adipocytes; this process is called adipogenesis. Mesodermal cells are influenced by various signals like bone morphogenetic proteins (BMPs), fibroblast growth factors (FGFs), transforming-growth factor β (TGFβ) and insulin like growth factor 1 (IGF1) to form preadipocytes (Lowe et al., 2011). Furthermore, preadipocytes undergo differentiation to mature adipocytes by several intracellular signaling molecules (Figure 2) including janus kinase-signal transducer and activator of transcription 3 (JAK-STAT3) (Zhang et al., 2011), glutathione (Vigilanza et al., 2011), SMAD proteins (Jin et al., 2006) and ribosomal protein S6 kinase 1 (S6K1) (Carnevalli et al., 2010) affecting adipogenic transcription factors. In preadipocytes, differentiation factors need to be released from their suppressive signaling molecules such as members of wingless (WNT) family (Ross et al., 2000), protein of the retinoblastoma (Rb) family (Scime et al., 2005), preadipocyte factor 1 (Pref1) (Smas and Sul, 1993) and Necdin, member of the melanoma-associated antigen family of proteins (Fujiwara et al., 2012) to undergo differentiation. The terminal differentiation to mature adipocytes is regulated by a number of transcriptional factors including early key regulator CAAT/enhancer binding proteins (C/EBPβ followed by C/EBPα, C/EBPδ), the master regulator PPARγ and sterol regulatory binding protein 1 (SREBP1) (Payne et al., 2009; White and Stephens, 2010). These transcriptional factors induce expression of various genes related to lipogenesis, lipolysis and insulin sensitivity including fatty acid binding protein (*FABP4*), lipoprotein lipase (*LPL*), glucose transporter (*GLUT4*) and fatty acid synthase (*FASN*) (Lefterova et al., 2008; Nielsen et al., 2008; Madsen et al., 2014).

Investigations of the molecular regulation of 1,25(OH)_2_D_3_ on adipogenesis have been conducted *in vitro*. In mouse 3T3-L1 preadipocytes, 1,25(OH)_2_D_3_ inhibits adipogenesis by acting on multiple targets suppressing C/EBPα and PPARγ expression, specifically antagonizing the transacting activity of PPARγ, and sequestering the nuclear receptor retinoic X receptor (RXR), a member nuclear receptor superfamily and down regulating both C/EBP β mRNA expression and C/EBP β nuclear protein levels (Kong and Li, 2006) (Figure 2). 1,25(OH)_2_D_3_ stimulates expression of the C/EBP β corepressor, eight twenty-one (ETO), and thus further inhibits the action of any remaining C/EBP β transcriptional effects required for adipogenesis (Blumberg et al., 2006).

Although early studies have established an inhibitory action of 1,25(OH)_2_D_3_ in 3T3-L1 preadipocytes differentiation, recently, a more specific effect of 1,25(OH)_2_D_3_ on WNT signaling emerged. WNT/β-catenin maintain preadipocytes in their undifferentiated state and thus preventing adipogenesis (Ross et al., 2000). The anti-adipogenic effect of 1,25(OH)_2_D_3_ is mediated by maintenance of WNT10B and nuclear β-catenin levels expression levels in 3T3-L1 preadipocytes, thereby suppressing transcription factor PPARγ (Lee et al., 2012). In addition, 1,25(OH)_2_D_3_ also inhibited mouse bone marrow stromal cells(BMSCs) differentiation into adipocytes by suppression of dickkopf1 (DKK1) and secreted frizzled-related protein 2 (SFRP2) expression levels via VDR mediated WNT signaling (Cianferotti and Demay, 2007).

In contrast, 1,25(OH)_2_D_3_ treatment of porcine mesenchymal stem cells (MSCs) stimulated both proliferation and differentiation in a dose dependent manner toward adipocytic phenotype by increasing PPARγ, LPL and adipocyte-binding protein 2 (AP2) mRNA levels (Mahajan and Stahl, 2009). In human tissue, 1,25(OH)_2_D_3_ promotes differentiation of already committed subcutaneous preadipocytes through increased expression of adipogenic markers *FABP4* and *LPL* (Nimitphong et al., 2012). Narvaez et al. (2013) demonstrated that mesenchymal cells differentiate in the presence of 1,25(OH)_2_D_3_ toward adipocytes with an enhanced lipid accumulation and increased expression of adipogenic marker genes (*FASN*, *FABP4*, and *PPARγ*).

In conclusion, 1,25(OH)_2_D_3_ regulates adipogenesis at various levels of the entire differentiation process (Figure 2). However, there are significant differences summarized in Table 1; the reasons for these differences are not clear at the moment—methodological differences as well as physiological roles of the adipose tissue in different species in their environments might affect these processes. Further studies are needed to address the effects of vitamin D in adipose tissue and its relevance in the associated diseases.

**Table 1:** **Effect of 1,25(OH)_2_D_3_ on adipogenesis in different species**.

**Species and cell type**	**Effect on adipogenesis**	**References**
**MOUSE**		
3T3-L1 preadipocytes	**Inhibition**	Blumberg et al., 2006; Kong and Li, 2006; Lee et al., 2012
	- *VDR* and *RXR* mediated suppression of *C/EBPα*, *PPARγ & C/EBPβ* (increased C/EBPβ corepressor ETO)	
	- Through maintenance of WNT10B and β-catenin levels	
Primary preadipocytes	**Promotion**	Nimitphong et al., 2012
	- Increasing *FABP4, adiponectin and PPARγ*	
Mouse bone marrow stromal cells(BMSCs)	**Inhibition**	Cianferotti and Demay, 2007
	- Suppression of DKK1 and SFRP2 (WNT suppressors)	
**PORCINE**		
Porcine preadipocytes	**Inhibition**	Zhuang et al., 2007
	- Inhibition of *PPARγ & RXR*, down regulated *LPL, PEPCK, GPDH, SCD1 & GLUT4*	
Porcine mesenchymal stem cells (MSCs)	**Promotion**	Mahajan and Stahl, 2009
	- Increased adipogenic markers (*PPARγ, LPL AP_2_*)	
**HUMAN**		
Subcutaneous preadipocytes	**Promotion**	Nimitphong et al., 2012
	- Increasing expression (*FABP4 & LPL*)	
Mesenchymal progenitor cells from human adipose tissue	**Promotion**	Narvaez et al., 2013
	- Increase of adipogenic marker genes (*FASN, FABP & PPARγ*)	

## Vitamin D and adipose tissue inflammation

In obesity, adipose tissue undergoes hypertrophic enlargement, which results in an imbalanced blood flow leading to hypoxia, inflammation and macrophage infiltration (Goossens, 2008; Trayhurn, 2013). The hypertrophied adipocytes are characterized by a reduced secretion of adiponectin and increased secretion of several proinflammatory cytokines such as interleukin IL-6, IL-8, TNF-α, resistin and MCP1 (Wellen and Hotamisligil, 2003; Maury and Brichard, 2010; Vlasova et al., 2010).

1,25(OH)_2_D_3_ acts at several levels to modulate the function of the immune system (Lemire, 2000). Several *in vitro* studies in the mouse 3T3-L1 cell line and human adipocytes have demonstrated that 1,25(OH)_2_D_3_ inhibits chronic inflammation in adipose tissue (Table 2). However, earlier studies performed in 3T3-L1 and human adipocytes demonstrate contradictory results favoring inflammatory cytokine expression (Sun and Zemel, 2008); the reasons for the contradictory findings are unclear. Recent evidence focuses on the involvement of 1,25(OH)_2_D_3_ in the regulation of adipose tissue inflammation by reducing the proinflammatory cytokines secreted from adipose tissue.

**Table 2:** **1,25(OH)_2_D_3_ and inflammation**.

**Cell type**	**1,25(OH)_2_D_3_ Mechanism of action**	**References**
Mouse 3T3-L1 and human adipocytes (differentiated from subcutaneous preadipocytes)	Increased IL-6 & TNFα in mouse 3T3-L1	Sun and Zemel, 2007
	Increased IL -6 and IL -8 in human adipocytes	
Mouse 3T3-L1 and human adipocytes (differentiated from subcutaneous preadipocytes)	Increased CD14, MIF, M-CSF, MIP, TNFα, IL -6, and MCP-1	Sun and Zemel, 2008
Human adipocytes (differentiated from subcutaneous preadipocytes)	Regulated nearly 140 genes favoring inflammation and oxidative stress	Sun et al., 2008
Mouse 3T3-L1 and Swiss mice on HFD supplemented with 1,25(OH)_2_D_3_	Reduction of IL -6 in both cell culture medium and tissue EFP	Lira et al., 2011
Preadipocytes isolated from human subcutaneous WAT	Reduction in MCP-1 and adiponectin	Lorente-Cebrian et al., 2012
Bone marrow-derived human mesenchymal stem cells and mature adipocytes from subcutaneous adipose tissue	Reduction in IL -6 and inhibited NF-κB nuclear translocation	Mutt et al., 2012
Mouse 3T3-L1 and human preadipocytes	Decreased IL -6, MCP-1, IL -1β and inactivation of NF-κB by inducing IκBα, decreased p38 phosphorylation	Marcotorchino et al., 2012
Human subcutaneous adipose tissue fragments	Reduction in MCP-1, IL -6, and IL -8.	Wamberg et al., 2013
Human preadipocytes	Reduction in MCP-1, IL -8 and IL -6 and inactivation of NF-κB by upregulation of IκBα	Gao et al., 2013
Human preadipocytes differentiated to mature adipocytes	Reduction in MCP1, IL -8, RANTES, IL -6 and IL -1β	Ding et al., 2013a,b
	Increased IκBα levels and reduced NF-κB p65 phosphorylation results in inhibition of NF-κB	
	Decreased phosphorylated p38 MAPK	

In differentiated adipocytes from human subcutaneous white adipose tissue 1,25(OH)_2_D_3_ attenuates TNF-α induced MCP-1 secretion, while it inhibited secretion of adiponectin without affecting its mRNA levels (Lorente-Cebrian et al., 2012). In human subcutaneous adipose tissue fragments 1,25(OH)_2_D_3_ reduced IL-1β induced expression of the inflammatory genes MCP-1, IL-6 and IL-8. However, results from the cell culture experiments have not been consistent with the *in vivo* findings. In a randomized controlled trial including fifty-five obese subjects, oral supplementation of vitamin D 7000 IU per day over 26 weeks did neither affect inflammation markers in the circulation nor in the adipose tissue (Wamberg et al., 2013). In mice on high fat diet, dietary supplementation of 1,25(OH)_2_D_3_ (0.05 mg/kg of diet) reduced their IL-6 protein content in epididymal adipose tissue and in the 3T3-L1 cell line stimulated by LPS (Lira et al., 2011).

Signal transduction of inflammatory pathways in adipose tissue involves activation of NF-κB and translocation of p65 to nucleus mediated by degradation of IκBα (Tourniaire et al., 2013). Mutt et al. (2012) have demonstrated that, 1,25(OH)_2_D_3_ suppressed LPS-stimulated IL-6 secretion in human isolated mature and MSC differentiated adipocytes. This was confirmed by Marcotorchino et al. (2012), who demonstrated that 1,25(OH)_2_D_3_ inhibits the inflammatory markers in both mouse and human adipocytes via the involvement of p38 MAP kinase and NF-κB classical inflammatory pathway and later by Gao et al. (2013) and Ding et al. (2013a).

**Figure 3:**
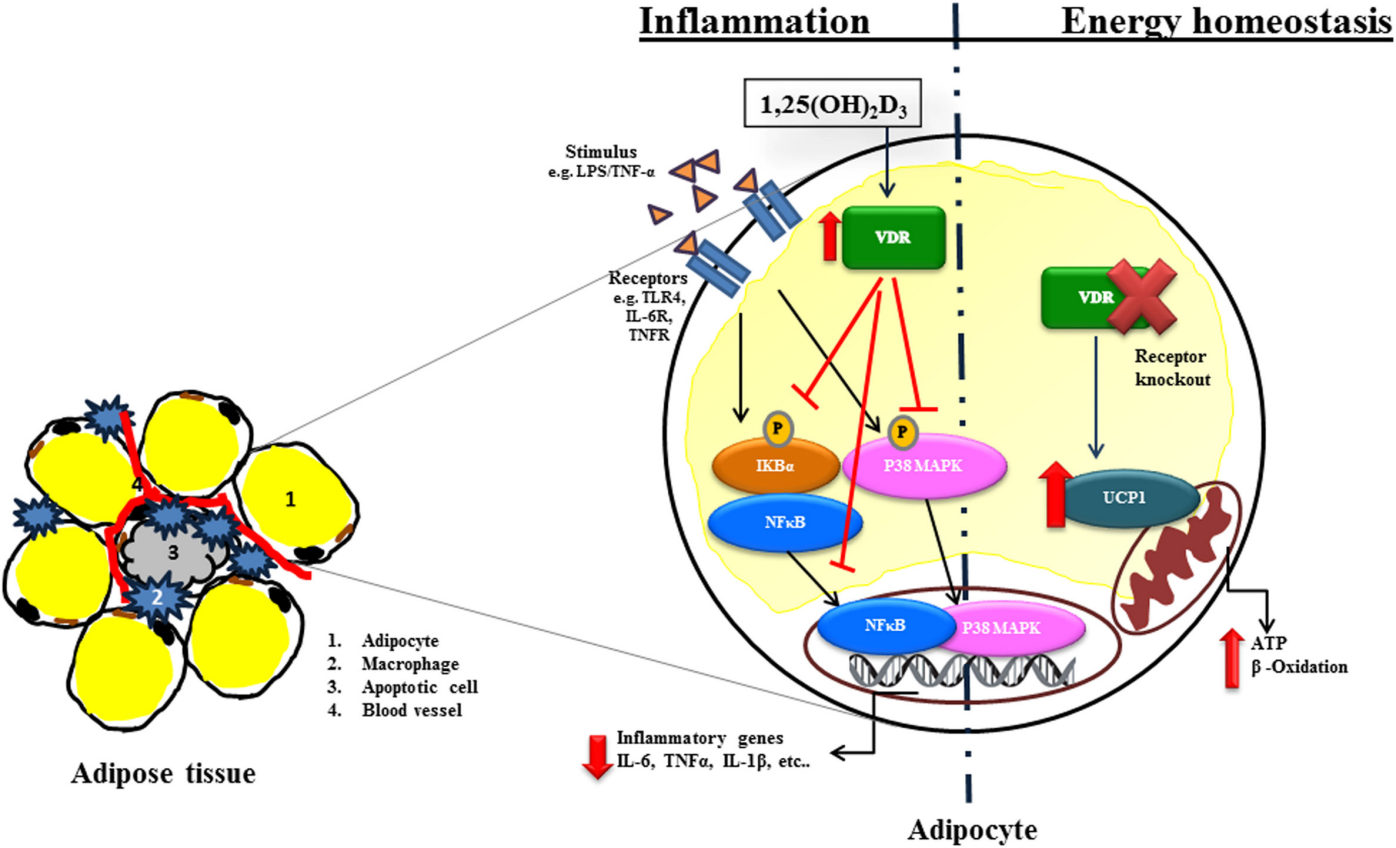
**Molecular actions of 1,25(OH)_2_D_3_ in inflammation and energy homeostasis in adipocytes**. Stimulation via e.g., lipopolysaccharide (LPS), TNF-α via specific receptors e.g., Toll like receptor (TLR), IL-6 receptors (IL-6R) activate Nuclear factor kappa-B (NFκB) or p38 mitogen-activated protein kinase (P38MAPK) signaling dependent transcription of inflammatory genes such as interleukin 6 (IL-6), tumor necrosis factor alpha (TNF-α) and interleukin 1 beta (IL-1β). 1,25(OH)_2_D_3_ inhibits inflammation by inhibiting Inhibitor kappa-B (IκBα) phosphorylation and translocation of NFκB as well P38MAPK into the nucleus. Furthermore, 1,25(OH)_2_D_3_ affects energy homeostasis. VDR^−/−^ mice increases energy expenditure through uncoupling proteins (UCPs). The arrows in the figure indicate activation and blunted line indicate inhibition. The red lines and arrows indicate the effect of 1,25(OH)_2_D_3_ on inflammatory signaling pathway.

In summary, the presence of 1,25(OH)_2_D_3_ inhibited chemokine and cytokine secretion in human adipocytes. 1,25(OH)_2_D_3_ strongly inhibited the activation of the NF-κB and MAPK signaling pathways, which prevent gene transcription of the proinflammatory factors (Figure 3). 1,25(OH)_2_D_3_ has been shown by different groups in different models to significantly reduce inflammation in the adipose tissue. However, further studies are needed to provide more evidence for the physiological relevance and the concentration levels of active 1,25(OH)_2_D_3_ in lean and obese subjects required to ameliorate the inflammation and associated complications.

## Vitamin D and adipose tissue energy homeostasis

The discovery of VDR expression in adipocytes was the cornerstone for the investigations of the effect of vitamin D on adipose tissue beyond its role in bone metabolism (Stumpf, 1995; Ding et al., 2012). Recent findings in genetically modified mouse models highlighted a new role for vitamin D and its receptor VDR in adipose tissue energy homeostasis. VDR knockout (VDR^−/−^) mice had reduced body weight and lower serum leptin concentrations, despite of an increased compensatory food intake compared to wild type mice of different genetic background C57BL6 and CD1. VDR^−/−^ mice were highly resistant to high fat diet induced weight gain (Narvaez et al., 2009). In addition, these mice are characterized by a relatively short lifespan, alopecia, osteoporosis, ectopic calcification, progressive loss of hearing and balance (Keisala et al., 2009; Tuohimaa, 2009). Mice lacking CYP27B1 [the (25(OH)D)-1α-hydroxylase enzyme, converts 25(OH)D_3_ in to 1,25(OH)_2_D_3_], displayed features similar to VDR^−/−^ with reduced body weight, hypoleptinemia and hyperphagia. Interestingly, uncoupling protein 1 (UCP-1) expression in white adipose tissue of the VDR^−/−^ mice was increased 25-fold.

In addition to reduced body weight, VDR^−/−^ mice had less body fat and lower levels of plasma triglycerides and cholesterol in comparison to the wild type counterparts even though mice were challenged with a high fat diet (Wong et al., 2009; Weber and Erben, 2013). The depletion of adipose tissue in younger VDR^−/−^ mice progresses with aging and resulted in severe mammary adipose tissue atrophy, along with the increased respiration and energy expenditure (Welsh et al., 2011). The effect on plasma lipid profile and unaltered food intake in these mice was confirmed by an increased β-oxidation rate in isolated adipocytes mediated by the induction of carnitine palmitoyltransferase II (CPTII) (Figure 3). VDR^−/−^ mice had an increased basal metabolism demonstrated by the total energy expenditure, oxygen consumption and CO_2_ production in comparison with the wild type mice (Wong et al., 2009). In addition, UCP1, UCP2, and UCP3 mRNAs were upregulated in brown adipose tissue of the VDR^−/−^ mice fed high fat diet. In contrast to VDR knock out models with the ablation of the receptor in the whole animal, adipose tissue specific overexpression of human VDR via the adipocyte fatty acid binding protein (aP2) promoter/enhancer element resulted in a decreased energy expenditure and oxygen consumption and thus the mice had an increased body weight and fat mass (Wong et al., 2011).

In conclusion, these transgenic animal models indicate a critical and complex role for 1,25(OH)_2_D_3_ and VDR signaling in energy homeostasis. However, notwithstanding the cell and mouse studies, further studies need to explore the role of vitamin D on human adipose tissue metabolism *in vivo*.

## Genetic view on the actions of VDR in adipocytes: integration with other tissues

The VDR genomic interactions in different types of cells and tissues have been mapped by *in vitro* experiments where target cells (primary or secondary) have been treated with 1,25(OH)_2_D_3_. Upon stimulation of VDR by its ligand, it forms a heterodimer with RXR and subsequently binds to the vitamin D response elements (VDREs) within the regulatory regions of target genes. The abundance of VDR binding sites and the regulation of changes in gene expressions are analyzed using array technology and the combination of chromatin immunoprecipitation (ChIP) with massive parallel sequencing (ChIP-seq). These advanced techniques have provided novel mechanistic insights of 1,25(OH)_2_D_3_ action via VDR in the regulation of cellular metabolism and disease states. However, studies on genome-wide actions of VDR in adipocytes are sparse.

Recent microarray studies of human adipocytes and preadipocytes incubated with macrophage-conditioned medium derived from U937 monocytes, confirmed the induction of genes associated with the metabolism and action of 1,25(OH)_2_D_3_, including CYP27B1 and VDR (Trayhurn et al., 2011). An earlier single microarray study in human subcutaneous adipose tissue derived preadipocytes differentiated to adipocytes demonstrated 237 1,25(OH)_2_D_3_ responsive genes (cell proliferation, angiogenesis, cell cycle, inflammation and response to oxidative stress) (Sun et al., 2008).

Most recent studies in the other cell types such as monocytes, primary CD4^+^ T-lymphocytes, adenocarcinoma, hepatic stellate and lymphoblastoid cell lines (LCLs) (Ramagopalan et al., 2010; Heikkinen et al., 2011; Meyer et al., 2012; Ding et al., 2013b; Handel et al., 2013; Tuoresmäki et al., 2014) contribute to a systemic understanding of 1,25(OH)_2_D_3_ induced gene regulation. Depending on the cell type, concentration and length of 1,25(OH)_2_D_3_ incubation approximately 2000 VDR genomic binding sites have been found in these studies. Yet, alterations in DNA accessibility in cell lines after short-term stimulation with 1,25(OH)_2_D_3_ may not reflect the physiological 1,25(OH)_2_D_3_ levels *in vivo* due to the different tissue environment and sympathetic influence. In primary CD4+ lymphocyte cells, isolated from nine healthy individuals with measured serum 25(OH)D levels, VDR binding sites ranged from 200 to 7118 across the genome and the corresponding 25(OH)D levels directly correlated with the number of VDR binding sites, suggesting far greater number of VDR binding sites in 1,25(OH)_2_D_3_ sufficient than the insufficient subjects (Handel et al., 2013).

Genome-wide VDR cistromes are not available in adipocytes, but recent VDR binding sites in other cell types has been mapped with ChIP-seq from both upstream and downstream of the transcription start site. Further genome wide view actions of VDR in adipocytes as well as integration of other tissue specific cell types are warranted.

## Conclusion and future directions

Adipose tissue acts in addition to nutrient storage as an active endocrine organ. In the obese state, sub-clinical inflammation increases the risk of a variety of chronic diseases. Vitamin D deficiency is common in overweight and obese individuals, and it is possible that lower circulating concentrations may contribute to increases in metabolic risk. A genome-wide association study of 25(OH)D concentrations in 33996 individuals of European descent from 15 cohorts found variants near genes involved in cholesterol synthesis, hydroxylation, and vitamin D transport affect vitamin D status (Wang et al., 2010). Genetic variation at these loci identifies individuals who have a substantially increased risk of vitamin D insufficiency.

On the cellular level, 1,25(OH)_2_D_3_ has a significant role in adipogenesis and inflammation which might be species dependent. Holick et al. (1989) demonstrated that the peak circulating concentrations of 25(OH)D in the elderly are about 30% of that of the young. These findings suggest that there will be significant challenges in the translation of the finding from models and non-human primates to the targeted human populations (healthy, diseased, black, white, age, BMI, geographical latitude, race). More evidence accumulates that one dose does not fit all (Powe et al., 2014). Powe and colleagues evaluated vitamin D binding proteins (VDBP) and 25(OH)D levels in black and white Americans. Black adult Americans had low 25(OH)D levels and with the threshold of 20 or 30 ng/ml, 77–96% of them would be classified as vitamin D deficient. Surprisingly, the black study participants had higher bone mineral density, higher calcium levels and only slightly higher parathyroid levels than the white study participants due to VDBP gene polymorphisms (rs7041 and rs4588). The authors speculated that the low levels of VDBP might protect against the adverse effects of vitamin D deficiency. Sufficient levels of this essential hormone and the development of potent novel vitamin D receptor analogs (Peräkylä et al., 2005; Leyssens et al., 2014), which could be easily and cheaply substituted, are beneficial in the maintenance of health and prevention of a number of diseases associated with vitamin D deficiency. Recent systemic review and meta-analysis summary of observational studies and randomized interventions investigated the association between the circulating 25(OH)D concentrations and cause specific mortality in 900,000 subjects in 26 countries (Chowdhury et al., 2014). There was an inverse association of mortality risk and vitamin D levels, yet the observed association could be direct [suboptimal 25(OH)D concentrations] or indirect through higher BMI or disadvantageous social circumstances. Thus, prospective intervention studies are needed to establish potential causal associations between vitamin D levels and disease outcomes.

### Conflict of interest statement

The authors declare that the research was conducted in the absence of any commercial or financial relationships that could be construed as a potential conflict of interest.

## Abbreviations

25(OH)D: 25-hydroxyvitamin D
1,25(OH)2D3: 1,25-dihydroxycholecalciferol
VDBPs: Vitamin D binding proteins
VDR: Vitamin D receptor
DKK1: dickkopf 1
SFRP2: Frizzled-related protein 2
BMSCs: bone marrow stromal cells
PPARγ: peroxisome proliferator-activated receptor gamma
RXRα: retinoid X receptor alpha
WNT10: wingless-type MMTV integration site family member 10
C/EBP (α, β, and γ): CCAAT/enhancer-binding proteins (α, β, and γ)
ETO: C/EBPβ corepressor eight twenty-one
FABP4: fatty acid binding protein 4
LPL: lipoprotein lipase
FASN: fatty acid synthase
SCD1: stearoyl-coA desaturase 1
GLUT4: glucose transporter type 4
PEPCK: phosphoenolpyruvate carboxykinase
LPS: lipopolysaccharide
TLR: toll like receptor
IL-6R: IL-6 receptors
NFκB: nuclear factor kappa-B
P38MAPK: p38 mitogen-activated protein kinase
IL-6: interleukin 6
TNF-α: tumor necrosis factor alpha
IL-1β: interleukin 1 beta
IκBα: inhibitor kappa-B
UCPs: uncoupling proteins
VDR ^−/−^: VDR knock out
CYP27B1^−/−^: CYP27B1 knock out
BMI: Body mass index
BMPs: bone morphogenetic proteins
FGFs: fibroblast growth factors
TGFβ: transforming-growth factor β
IGF1: insulin like growth factor 1
JAK-STAT3: janus kinase-signal transducer and activator of transcription 3
S6K1: ribosomal protein S6 kinase 1
WNT: wingless family
Rb: protein of retinoblastoma family
Pref1: preadipocyte factor 1
Necdin: melanoma-associated antigen family of proteins member
SREBP1: sterol regulatory binding protein 1
MSCs: mesenchymal stem cells
AP2: adipocyte-binding protein 2
MCP1: monocyte chemoattractant protein 1
CYP27B1: (25(OH)D)-1α-hydroxylase
CPTII: carnitine palmitoyltransferase II
WAT: White adipose tissue
VDREs: vitamin D response elements
ChIP-seq: chromatin immunoprecipitation—sequencing
LCLs: lymphoblastoid cell lines
